# Supplementary material for: Contribution of RND-Type Efflux Pumps in Reduced Susceptibility to Biocides in Acinetobacter baumannii
Source: Antibiotics (Basel). 2022 Nov 16;11(11):1635. doi: 10.3390/antibiotics11111635 (PMC9686468; doi:10.3390/antibiotics11111635)
Supplement: Supplementary file 1 [file antibiotics-11-01635-s001.zip › antibiotics-2019482-supplementary.pdf]

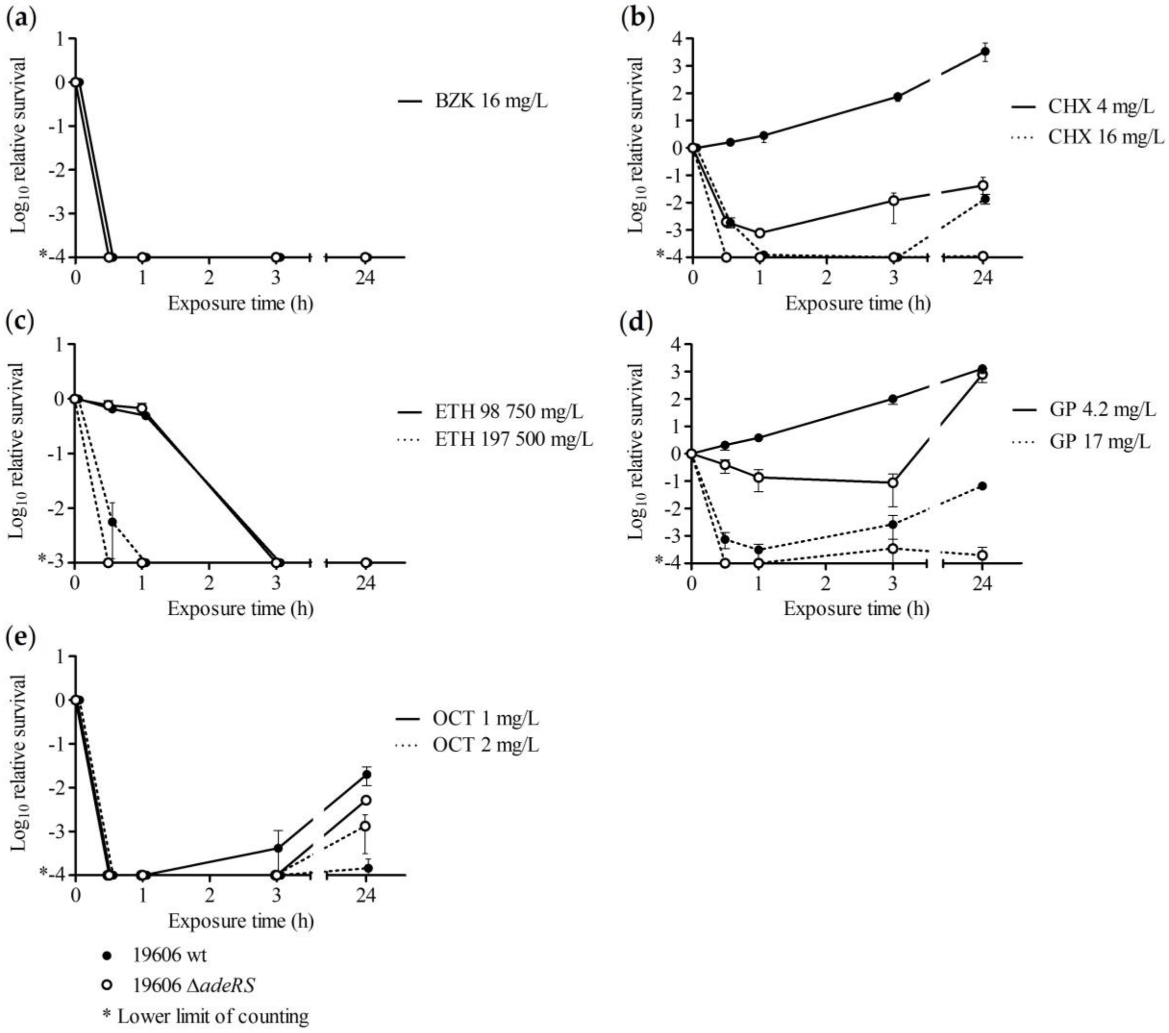

Figure S1. Time-kill curves for *A. baumannii* ATCC 19606 wt and 19606 Δ*adeRS* (*adeABC* not expressed) with additional biocide concentrations: (**a**) Benzalkonium chloride (BZK), (**b**) Chlorhexidine digluconate (CHX), (**c**) Glucoprotamin (GP), (**d**) Ethanol (ETH), (**e**) Octenidine dihydrochloride (OCT). Error bars represent the range from three independent experiments.

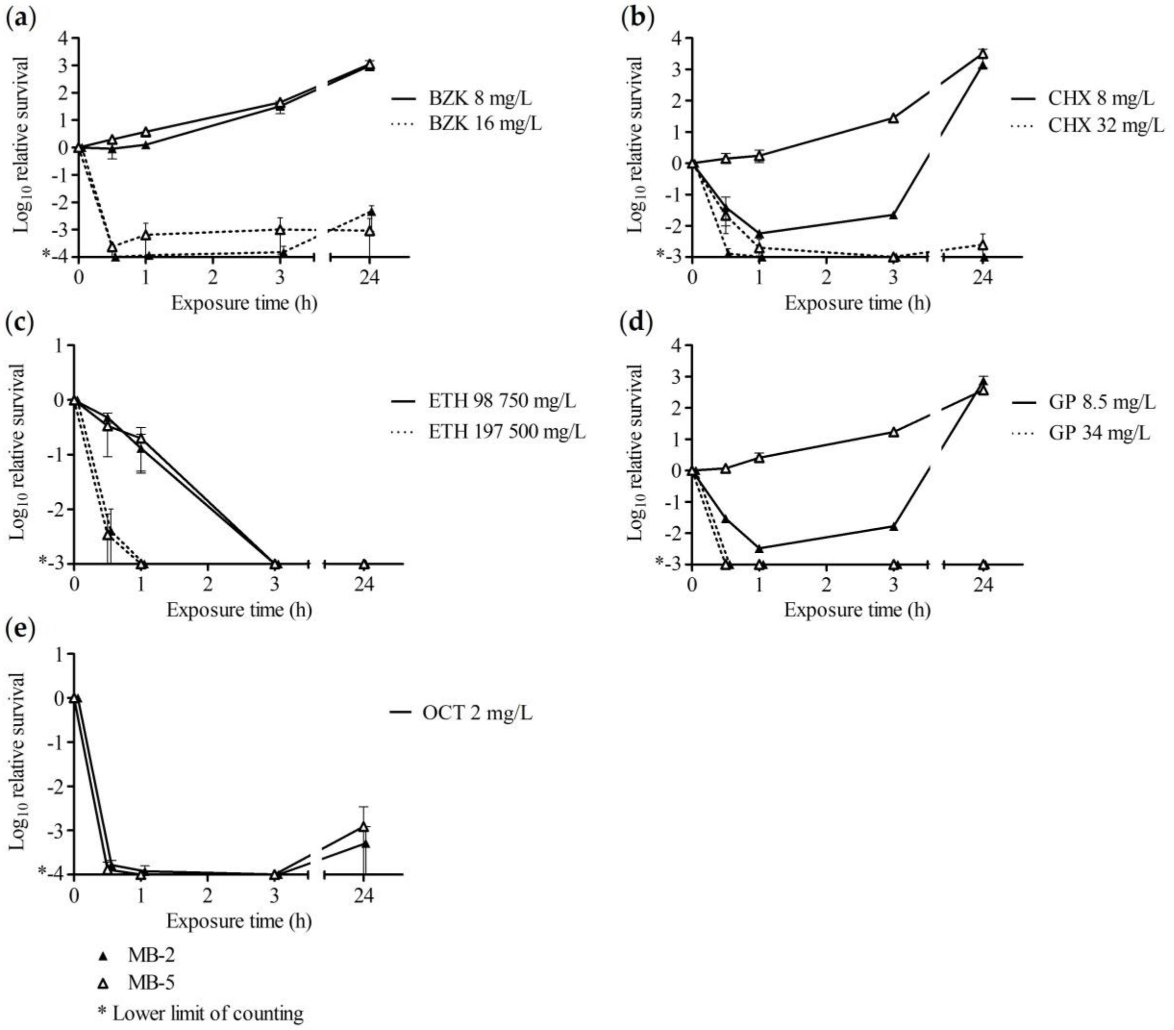

Figure S2. Time-kill curves for *A. baumannii* MB-2 and MB-5 (*adeABC* overexpressed) with remaining biocide concentrations: (**a**) BZK, (**b**) CHX, (**c**) GP, (**d**) ETH, (**e**) OCT. Error bars represent the range from three independent experiments.

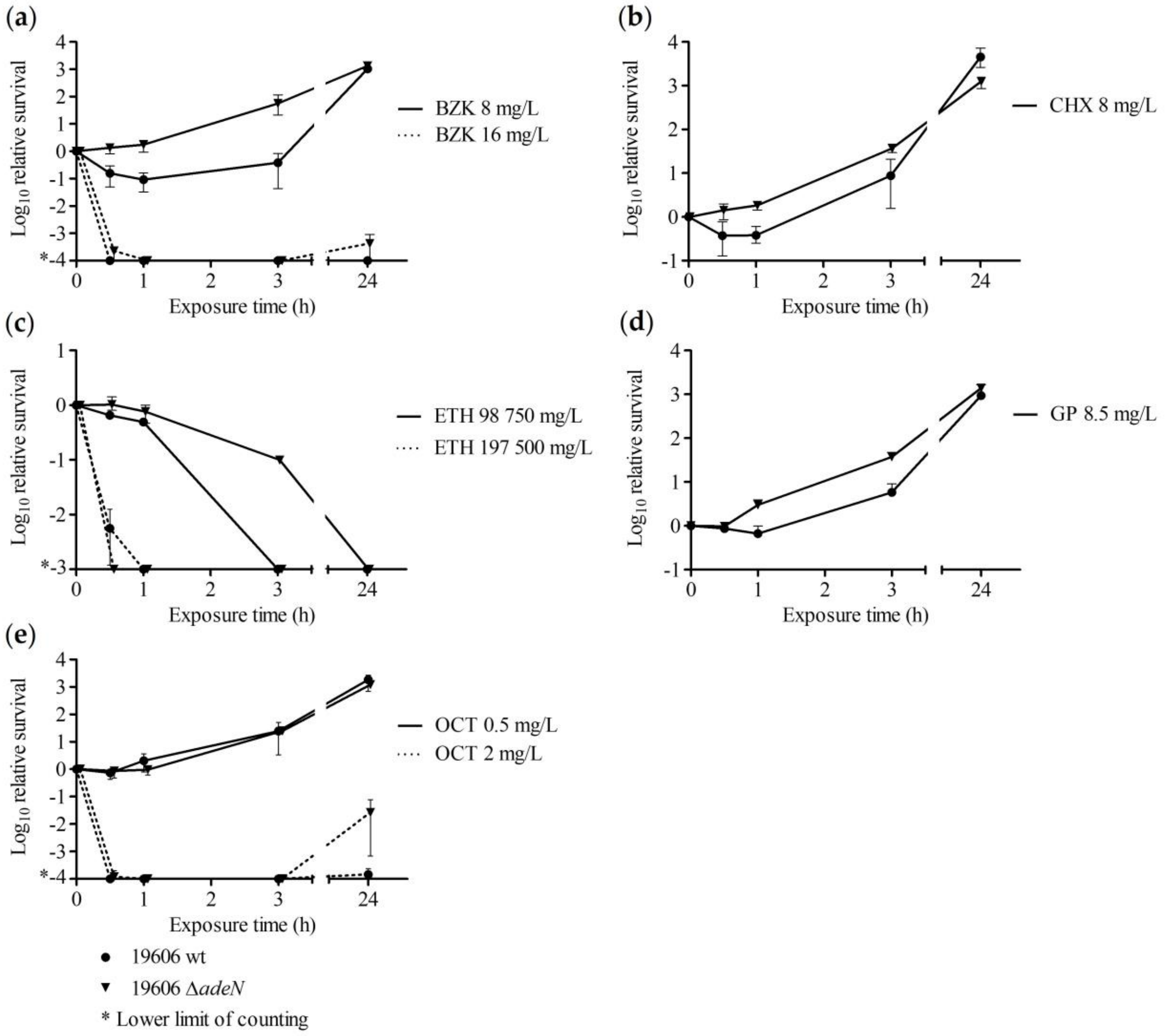

Figure S3. Time-kill curves for *A. baumannii* ATCC 19606 wt and 19606 Δ*adeN* (*adeIJK* overexpressed) with additional biocide concentrations: (**a**) BZK, (**b**) CHX, (**c**) GP, (**d**) ETH, (**e**) OCT. Error bars represent the range from three independent experiments.

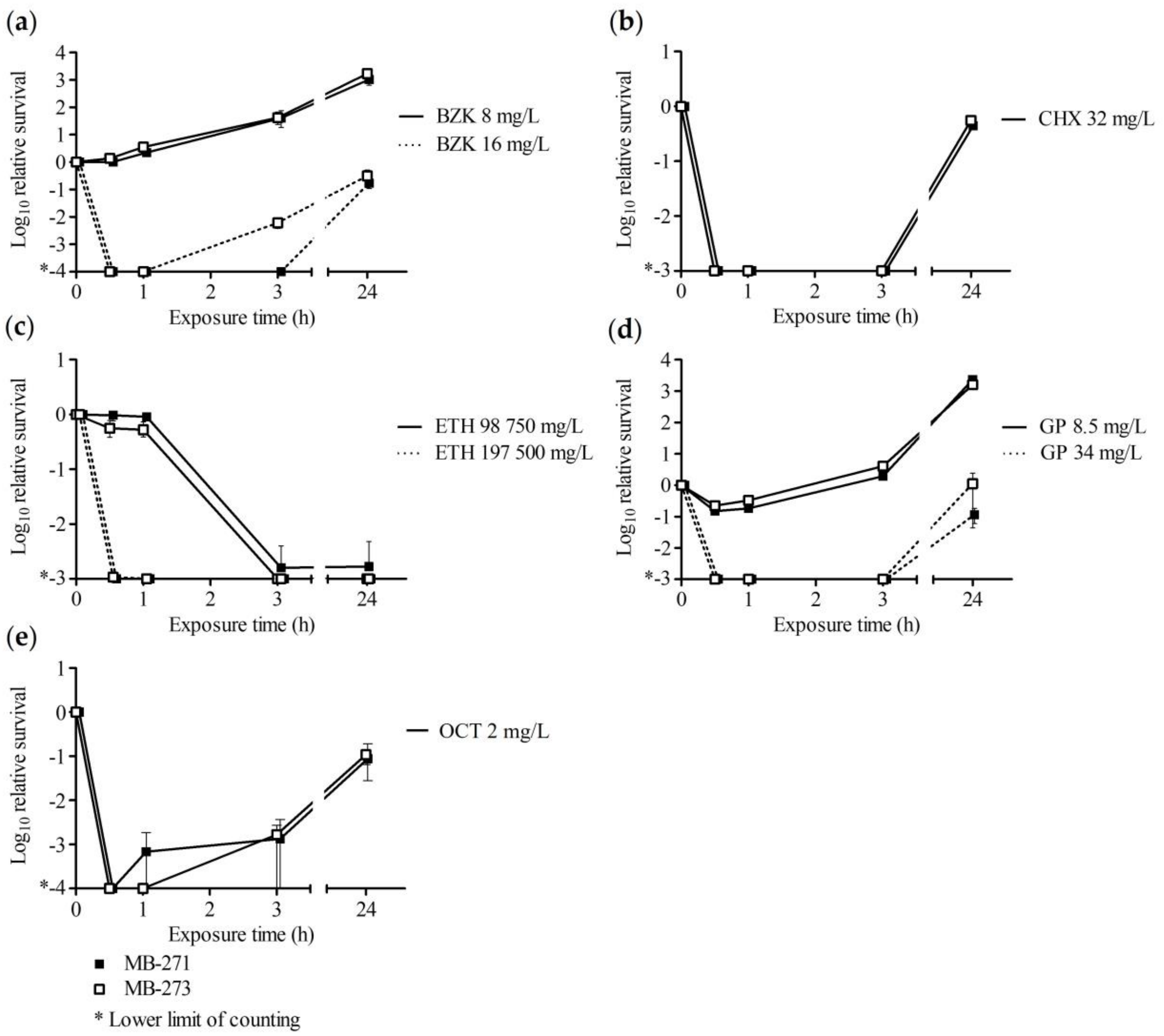

Figure S4. Time-kill curves for *A. baumannii* MB-271 and MB-273 (*adeIJK* overexpressed) with remaining biocide concentrations: (**a**) BZK, (**b**) CHX, (**c**) GP, (**d**) ETH, (**e**) OCT. Error bars represent the range from three independent experiments.

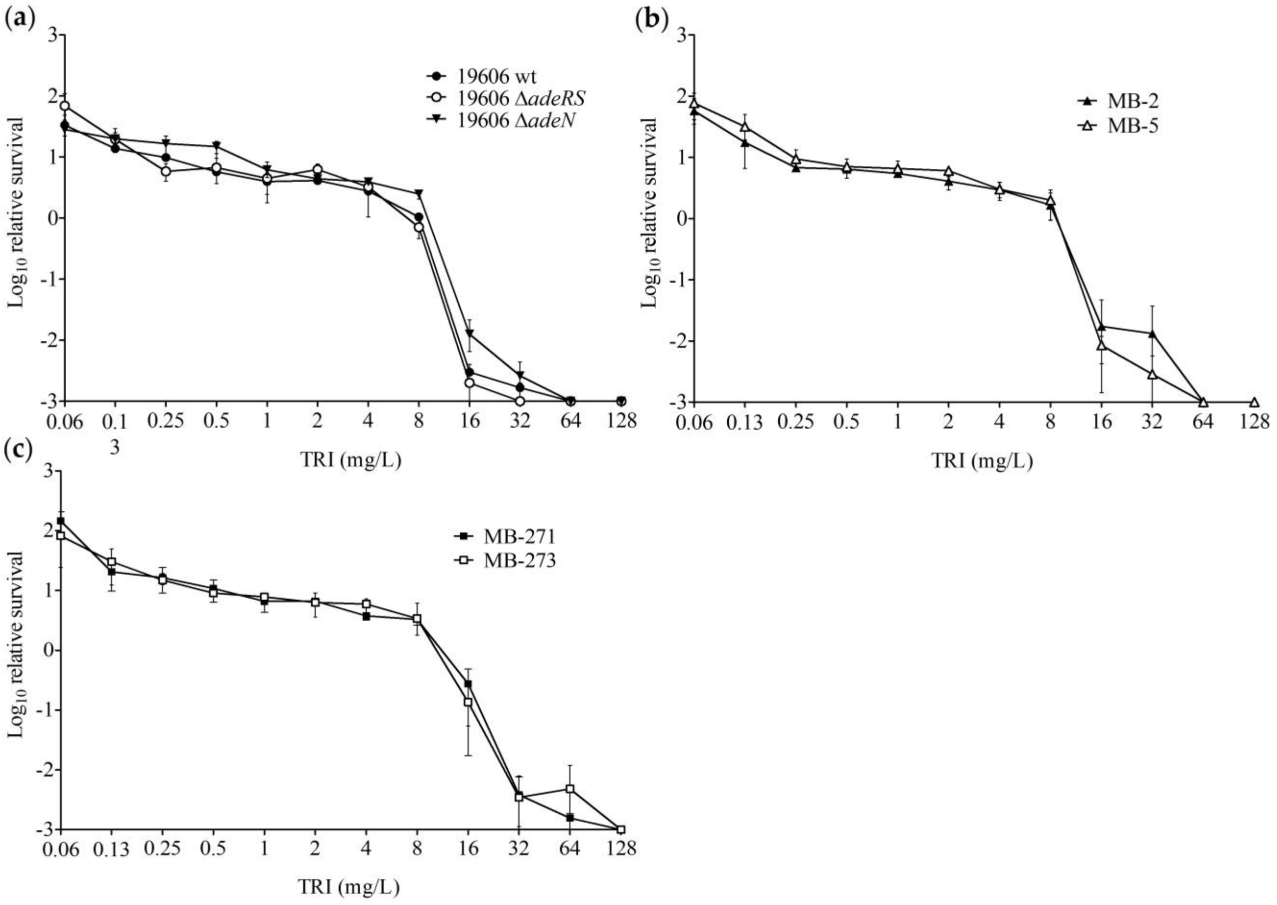

Figure S5. Dose response of *A. baumannii* regulatory system mutant strains to triclosan (TRI): (**a**) for *A. baumannii* ATCC 19606, *A. baumannii* ATCC 19606 Δ*adeRS* and *A. baumannii* ATCC 19606 Δ*adeN*, (**b**) for *A. baumannii* MB-2 and *A. baumannii* MB-5, (**c**) for *A. baumannii* MB-271 and *A. baumannii* MB-273. Error bars represent the range from three independent experiments.

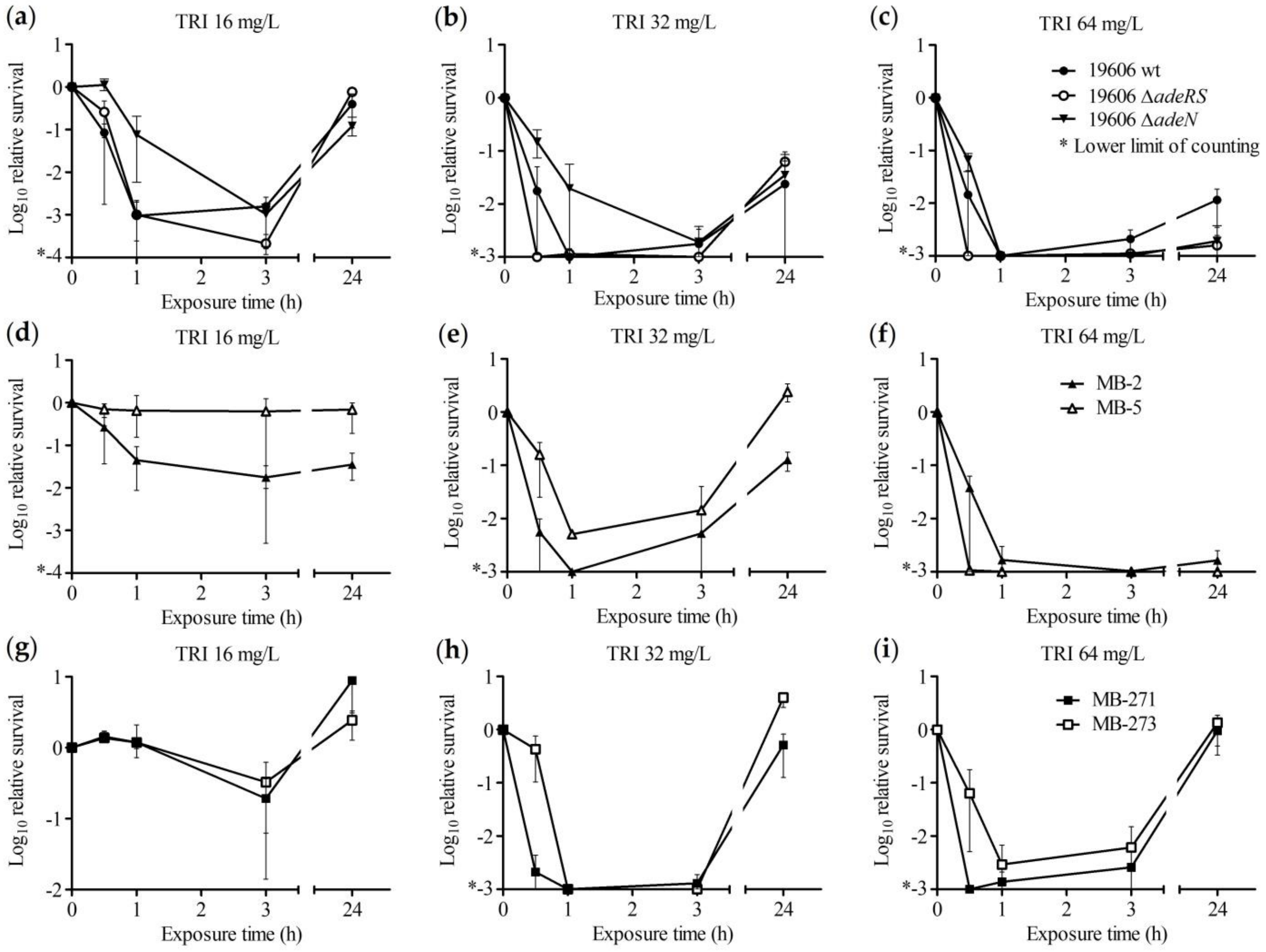

Figure S6. Time-kill curves with different TRI concentrations: (**a**) – (**c**) for *A. baumannii* ATCC 19606, *A. baumannii* ATCC 19606 Δ*adeRS* and *A. baumannii* ATCC 19606 Δ*adeN*, (**d**) – (**f**) for *A. baumannii* MB-2 and *A. baumannii* MB-5, (**g**) – (**i**) for *A. baumannii* MB-271 and *A. baumannii* MB-273. Error bars represent the range from three independent experiments. * Lower limit of counting.
